# *Baeopterogyna mihalyii* Matile (Diptera, Mycetophilidae): association of sexes using morphological and molecular approaches with the first description of females

**DOI:** 10.3897/zookeys.114.1364

**Published:** 2011-06-30

**Authors:** Olavi Kurina, Erki Õunap, Gordon Ramel

**Affiliations:** 1 Institute of Agricultural and Environmental Sciences, Estonian University of Life Sciences, Riia st 181, 51014 Tartu, Estonia; 2 Institute of Ecology and Earth Sciences, University of Tartu, Vanemuise 46, EE-51014 Tartu, Estonia; 3 Museum of Zoology, University of Tartu, Vanemuise 46, EE-51014 Tartu, Estonia; 4 Ploy Plu Residency, 165/5 Moo 9, Pho Khum Rd., Rob Wiang Sub-District, Muang District, Chiang Rai. 57000, Thailand

**Keywords:** Diptera, Mycetophilidae, *Baeopterogyna*, systematics, Europe, COI

## Abstract

Both males and females of *Baeopterogyna mihalyii*Matile, 1975 are recorded from northern Greece. Females are described for the first time providing photographs of the general facies and terminalia. In contrast to the single congener with stenopterous females – *Baeopterogyna nudipes* Vockeroth, 1972 – *Baeopterogyna mihalyii* is shown to have normally developed wings in both sexes. Association of sexes is based on both morphological characters and sequence data from cytochrome oxidase subunit one (COI). DNA sequences are used for the first time for the association of sexes in Mycetophilidae.

## Introduction

*Baeopterogyna* Vockeroth, 1972 is a small genus of Mycetophilidae including only two species: *Baeopterogyna nudipes* Vockeroth, 1972 from the Nearctic and *Baeopterogyna mihalyii*Matile, 1975 from the Palaearctic region, respectively. The genus belongs to the subfamily Sciophilinae and is most closely related to the *Neuratelia* Rondani (Vockeroth 1972, Matile 1975a) distinguished from it by absence of distinct tibial bristles, setosity of the thorax and wing (Søli et al. 2000), and structure of the male terminalia. Both known species of *Baeopterogyna* have simple slender gonostyli compared to the complex convoluted gonostyli of *Neuratelia*. The type species – *Baeopterogyna nudipes* – was described by Vockeroth (1972) from North America (Yukon Territory and Alaska) but since then it has not been recorded. The European species – *Baeopterogyna mihalyii* – was described from Hungarian material (Matile 1975a) and subsequently it has been recorded in the Czech and Slovak Republics (Ševčík 1999, Chandler 2005). Females of *Baeopterogyna nudipes* are described as stenopterous (Vockeroth 1972) while females of *Baeopterogyna mihalyii* were so far unknown but, analogically to the congener, were also supposed to have reduced wings (e.g. Søli et al. 2000).

Since the introduction of the ‘DNA barcoding’ approach (Hebert et al. 2003), it has repeatedly been shown for different groups of organisms that the intraspecific genetic distances of the mitochondrial *COI* gene are in most cases at least a magnitude smaller than the interspecific genetic distances. Moreover, it has been demonstrated that sharing the same *COI* haplotype between different species is rare (Hebert et al. 2010, Raupach et al. 2010, Hausmann et al. 2011). Exceptions to these rules are not frequent and it has been shown that ambiguities have often been derived from taxonomically poorly known groups (Handfield and Handfield 2006; Ståhls and Savolainen 2008, Alexander et al. 2009). These findings have allowed the use of *COI* sequences in different approaches. In addition to being useful in identifying and delimiting species that are otherwise hard to distinguish (Janzen et al. 2005, Burns et al. 2008, Pauls et al. 2010), these so-called ‘DNA barcodes’ have been shown to be powerful tools in e.g. clarifying the status of morphologically different sexes or races of polymorphic species (Foottit et al. 2009, Janzen et al. 2009) and associating different life stages of insects (Zhou et al. 2007, Pauls et al. 2010). These two practices have repeatedly been implemented in earlier studies of Diptera (e.g. Carew et al. 2005, Ekrem et al. 2010, Stur and Ekrem 2011), but to the best of our knowledge, no attempts have been made to utilize them in research of Mycetophilidae. In this article, we implemented section from the 3’ end of the *COI* gene, commonly used in the phylogenetic studies of the Mycetophilidae (Rindal et al. 2007, 2009). Despite this part of the *COI* does not overlap with the so-called ‚barcoding fragment’ from near the 5’ end of the gene, it still is influenced by the identical evolutionary processes as part of the same gene. Therefore, all predictions and conclusions regarding to the systematic utility of the ‚barcoding fragment’ can be attributed to the 3’ end of *COI*.

The current study was initiated by finding both sexes of *Baeopterogyna mihalyii* in Malaise trap samples from northern Greece. The aims of this article are to describe the so far unknown female of *Baeopterogyna mihalyii* and introduce a possibility of using *COI* sequence data for association of females and males of fungus gnat species.

## Material and methods

### Collection, illustration and morphological study

All *Baeopterogyna mihalyii* material was collected by GR from the Kerkini Lake area in Northern Greece south of the Bulgarian border. Despite an extensive Mycetophilidae material collected from the area during a survey of invertebrates from 2003 to 2009 (for details see Ramel et al. 2008 and http://www.ramel.org/lake-kerkini/project.html), only samples from one home-made Malaise trap yielded 11 specimens of *Baeopterogyna mihalyii*. The trap was situated over the bog from where the Sultanitsa stream springs and faced down hill into a beech (*Fagus sylvatica*) forest. All material was initially collected and preserved in 70% ethanol while the final preservation method of the studied specimens is indicated in Table 1. For detailed study of terminalia they were detached and cleared in solution of KOH, followed by neutralization in acetic acid and washing in distilled water (see also Kurina 2003). The remaining chitinous parts were either (1) inserted into glycerine for study and photography and thereafter preserved as glycerine preparations in polyethylene micro vials or (2) slide-mounted in Euparal following the method described by Kurina (2008). After detaching the terminalia, the remaining part of the abdomen was used for molecular study while the rest of body was slide-mounted. Some male specimens are preserved in alcohol or dry-mounted in accordance with the method dercribed by Vockeroth (1966). The habitus photos were taken in alcohol using a Canon EOS7D camera fitted with a Canon MP-E65 (F2.8 1–5 x) lens. Illustrations of male and female terminalia are combined using Helicon Focus 4.7 software, from several partly focused images taken with a Leica DFC295 camera attached to an Olympus CX31 compound microscope. Morphological terminology follows Søli (1997).

The material has been deposited in IZBE (Institute of Agricultural and Environmental Science, Estonian University of Life Sciences, former Institute of Zoology and Botany) and all specimen data have been inserted into the database of Estonian animal collections (Abarenkov et al. 2010, see also http://elurikkus.ut.ee/collections.php?lang=eng). All specimen information including photographs is available also on the Fungus Gnats Online website (www.sciaroidea.info/taxonomy/45717).

### Molecular techniques

The genomic DNA was extracted using a High Pure PCR Template Preparation Kit (Roche Diagnostics GmbH, Mannheim, Germany). Anterior segments of the abdomen that had been stored after genitalia dissection were crushed and used for the extraction. This process was carried out following the manufacturer’s instructions for extraction of genetic material from mammalian tissue.

A 762-bp fragment of cytochrome C oxidase subunit 1 (*COI*), corresponding to positions 2228–2989 of the mitochondrial genome of *Drosophila melanogaster* Meigen, 1830 (RefSeq NC_001709) was amplified and sequenced using primers C1-J-2195 (5’-TTGATTTTTTGGTCACCCTGAAGT-3’) and TL2-N-3014 (5’-TCCAATGCACTAATCTGCCATATTA-3’) (Simon et al. 1994). PCR was performed in a total volume of 20 µl, with the reaction mixture containing 1X BD Advantage 2 PCR buffer, 1U BD Advantage 2 Polymerase mix (BD Biosciences, San Jose, USA), 0.2 mM dNTP (Fermentas, Vilnius, Lithuania), 4 pmol of primers and 20–80 ng of purified genomic DNA. PCR was carried out in a Biometra T1 Thermocycler (Biometra, Göttingen, Germany), its conditions were an initial denaturation at 94°C for 2 min, 35 cycles of 30 s at 94°C, 30 s at 50°C and 1 min at 68°C, followed by a final extension at 68°C for 7 min. PCR products were visualised on a 1.6% agarose gel, and 10 μl of the PCR solution was treated with fast alkaline phosphatase and exonuclease I (Fermentas). DNA cycle sequencing was performed in a total volume of 10 μl using the Big Dye Terminator v.3.1 Cycle Sequencing Kit (Applied Biosystems, Foster City, USA). Cycling conditions were: initial denaturation for 1 min at 96°C followed by 25 cycles of 10 s at 95°C, 15 s at 47°C and 4 min at 60°C. Both DNA strands were sequenced using 1.6 pmol of primers. The sequences were resolved on a 3730xl DNA Analyzer (Applied Biosystems).

### Phylogenetic analysis

In total, 9 specimens including three species of fungus gnats from the subfamily Sciophilinae were analysed. In addition to 3 males and 2 females of *Baeopterogyna mihalyii*, both sexes of *Allocotocera pulchella* (Curtis 1837) and both sexes of *Sciophila nigronitida* Landrock, 1925, the latter as an outgroup, were included. For detailed information about specimens see Table 1.

Consensus sequences were created with the program Consed (Gordon et al. 1998) using sequence data from both DNA strands. Sequences were double-checked by eye and aligned with ClustalW (Thompson et al. 1994), using BioEdit (Hall 1999) as a sequence editor. Modeltest 3.06 (Posada and Crandall 1998) was used to search for the optimal model of DNA substitution. Bayesian phylogenetic inference, maximum likelihood (ML), maximum parsimony (MP) and neighbour-joining (NJ) approaches were all used to evaluate the robustness of the phylogenetic analysis. The GTR+I model, selected by Modeltest using Akaike Information Criterion, was implemented for NJ and ML analysis in PAUP*4.0b10 (Swofford 1998). Branch supports were assessed using 1000 bootstrap replicates. MP analysis with simple addition of taxa was also performed in PAUP and resulted in a single most parsimonious tree. Branch supports for this tree were assessed using 1000 bootstrap replicates, with 10 heuristic searches and simple addition of taxa used for each replicate. ML, NJ and MP trees were visualised in TreeView 1.6.6 (Page 1996).

Bayesian phylogenetic analysis implementing the GTR+I model was performed using MrBayes 3.1 (Ronquist and Huelsenbeck 2003). Four simultaneous Markov chains (one cold and three heated) were run for 4 million generations, with trees sampled every 1000 generations. Likelihood values were inspected, and the first 1000 sampled trees were discarded as ‘burn-in’. To estimate posterior probabilities of recovered branches, a 50% majority rule was applied. Phylograms were created as average-branch-length consensus trees and visualised in TreeView 1.6.6**.**

## Results and discussion

For determination of male material of *Baeopterogyna mihalyii*, the key to mycetophilid genera by Søli et al. (2000) was used successfully. In addition, male specimens were compared with type material of *Baeopterogyna nudipes* (paratypes, 2♂♂, in MNHN; see also Vockeroth 1972) and they were found to be congeneric. Primary association of sexes of *Baeopterogyna mihalyii* was based on simultaneous finding of females and males that were morphologically similar, except characters in terminalia, in the same sample. However, these females have normally developed wings while those of *Baeopterogyna nudipes* are stenoperous (Matile 1975a, Søli et al. 2000). Moreover, they lack setae on the upper part of the anepisternum which are present in males, a character that is considered diagnostic of the genus by Søli et al. (2000). Therefore, additional support by COI sequence data was needed to associate the sexes of *Baeopterogyna mihaylii* unambiguously.

**Figure 1.**
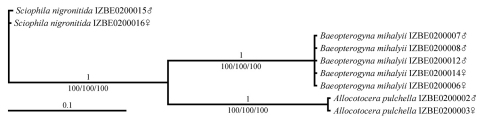
Bayesian phylogenetic tree (GTR+I model) of selected Mycetophilidae taxa, based on a 762 bp fragment of a *COI* gene. Bayesian posterior probabilities are given above the branches; bootstrap support for the ML/NJ/MP trees, which exhibited identical topology, are presented below the branches.

All specimens identified preliminarily as *Baeopterogyna mihalyii* according to their morphological characteristics carried identical COI haplotypes, and the same applied for both *Allocotocera pulchella* individuals, thus proving that morphology-based identification was correct. The *Sciophila nigronitida* specimens, however, carried different COI haplotypes at one locus corresponding to position 2508 of the full mitochondrial genome of *Drosophila melanogaster* (RefSeq NC_001709); the male had an adenine nucleotide, whereas the female had a guanine nucleotide. Since the genetic distance between these two specimens is only 0,13%, i. e. significantly below the average pairwise distance between individuals belonging to different species (Hebert et al. 2010, Raupach et al. 2010, Hausmann et al. 2011), we conclude that their conspecificity is not under question. Due to the non-existing or minimal genetic distance between the conspecific individuals, all three species formed clearly monophyletic well-supported lineages in the phylogenetic trees (Figure 1). Concerning the main question of the current article, we conclude that it has been proven that the hitherto unknown females of *Baeopterogyna mihalyii* have fully developed wings in contrast to the stenopterous females of its only known congener, *Baeopterogyna nudipes*.

Among the species used for phylogenetic analysis also *Sciophila nigronitida* is representing the first record from Greece (for collecting details see Table 1).

**Table 1.** Details of specimens used for taxonomic study and molecular analysis

Voucher No	Species	Sex	Collecting site, collecting method and collector	Date	Method of preservation	GenBank acc. code for COI
IZBE0200002	*Allocotocera pulchella* (Curtis, 1837)	♂	Estonia, Palupõhja 58°25'54.68"N 26°14'28.90"E, Malaise trap, Soon, V. leg.	25.vii – 4. viii 2009	Abdomen used for DNA sequencing; terminalia in glycerin; rest of body dry mounted from ethanol	JN007851
IZBE0200003		♀			Abdomen used for DNA sequencing; terminalia in glycerin; rest of body dry mounted from ethanol	JN007851
IZBE0200004	*Baeopterogyna mihalyii* Matile, 1975	♂	Greece, Central Macedonia, Kerkini lakes area, village Neo Petritsi, Sultanitsa site, 41°19'02.1"N 023°12'05.0"E, 1485 m a.s.l., Malaise trap, Ramel G. leg.	12 – 18.v 2008	In ethanol	
IZBE0200005		♂			In ethanol	
IZBE0200006		♀			Abdomen used for DNA sequencing; terminalia in glycerin; rest of body in ethanol	JN007850
IZBE0200007		♂		19 – 25. v 2008	Abdomen used for DNA sequencing; rest of body and terminalia slide mounted	JN007850
IZBE0200008		♂			Abdomen used for DNA sequencing; terminalia in glycerin; rest of body in ethanol	JN007850
IZBE0200009		♂			Slide mounted under 5 different coverslips	
IZBE0200010		♂			In ethanol	
IZBE0200011		♂			In ethanol	
IZBE0200012		♂		25.v – 1. vi 2008	Abdomen used for DNA sequencing; rest of body and terminalia slide mounted	JN007850
IZBE0200013		♂			In ethanol	
IZBE0200014		♀			Abdomen used for DNA sequencing; terminalia in glycerin; rest of body slide mounted	JN007850
IZBE0200015	*Sciophila nigronitida* Landrock, 1925	♂	Greece, Central Macedonia, Kerkini lakes area, village Neo Petritsi, Farfara site, 41°19'30.5"N 023°15'00.1"E, 750 m a.s.l., Malaise trap, Ramel G. leg.	16 – 22. vi 2008	Abdomen used for DNA sequencing; terminalia in glycerin; rest of body dry mounted from ethanol	JN007853
IZBE0200016		♀			Abdomen used for DNA sequencing; terminalia in glycerin; rest of body dry mounted from ethanol	JN007852

## Taxonomy

### Baeopterogyna mihalyii

Matile, 1975

http://species-id.net/wiki/Baeopterogyna_mihalyii

Figs 2 3

#### Material studied:

9♂♂ 2♀♀, for collecting data see Table 1: voucher numbers from IZBE0200004 to IZBE0200014.

**Figure 2.**
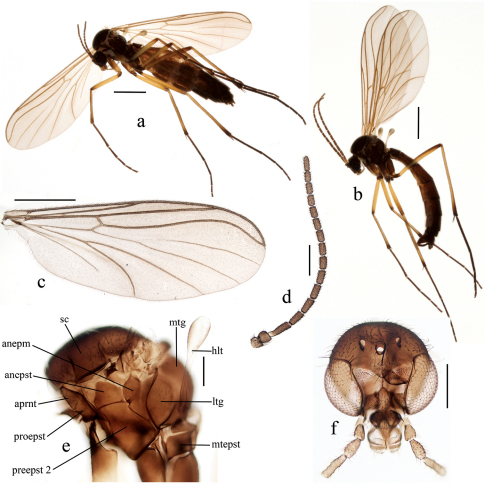
*Baeopterogyna mihalyii*. **a** female **b** male **c** female wing **d** female antenna **e** female thorax **f** female head (last palpal segments absent). Scale = 1 mm (a, b, c), 0.2 mm (d, e, f).<br/> *anepm = anepimeron; anepst = anepisternum; aprnt = antepronotum; htl = halter; ltg = laterotergite; mtepst = metepisternum; mtg = mediotergite; proepst = proepisternum; preepst = preepisternum; sc = scutum*.

**Figure 3.**
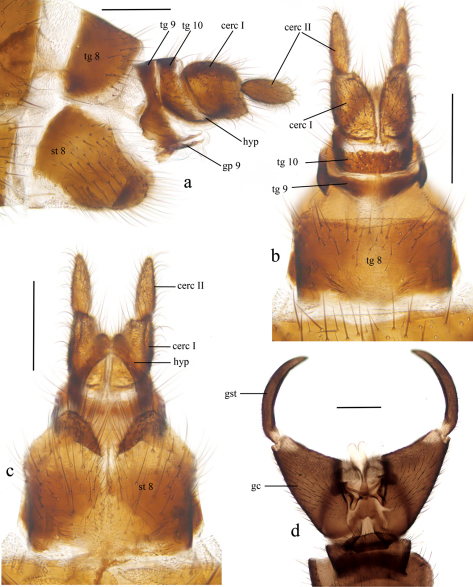
*Baeopterogyna mihalyii*. **a** female terminalia, lateral view **b** female terminalia, dorsal view **c** female terminalia, ventral view **d** male terminalia, ventral view. Scale = 0.2 mm.<br/> *cerc = cercus; gc = gonocoxite; gp = gonapophysis; gst = gonostylus; hyp = hypoproct; st = sternite; tg = tergite*

Female (Figs 2a, 2c – 2f, 3a – 3c).

#### Description.

Length of body 4.65 – 4.94 mm (n=2).

Head brown with dark setae. Three equally sized ocelli in a triangular arrangement. Clypeus subrounded. Palpus with 5 light brown setose segments with ratios of 1: 1.17:1.58: 2.25:4.33. Mouthparts brownish. Antenna with 2+14 segments. Scape, pedicel and base of first flagellar segment light brown, rest of flagellomeres brown. Scape with sparse setae including dorsoapicals extending to middle of pedicel. Pedicel with sparse and all flagellomeres with dense setae. First flagellomere 3 times as long as wide, succeeding segments gradually shorter. Apical flagellomere cylindrical, about three times as long as wide.

Thorax brown. Scutum covered with pale setae including long lateral hairs. Lateral parts of thorax slightly paler than scutum. Antepronotum with numerous long pale hairs. Proepisternum with numerous shorter setae. Laterotergite and mediotergite with upward directed hairs. Anepimeron and metepisternum with short setae, anepisternum bare. Scutellum with setae not in distinct pairs.

Legs. Fore coxa light brown with hind margin and apical fourth yellow. Mid and hind coxae brown, apically slightly paler. All trochanters brown. All femora and tibiae yellow with apical brown markings. All tarsi dark brown. Tibiae with irregularly arranged setae but without distinct bristles. Fore tibia with a spur 0.18 of basitarsus length. Mid and hind tibiae both with two equal spurs, 0.19 and 0.21 of basitarsus length, respectively. Ratio of femur to tibia for fore, mid and hind legs: 1.00; 0.82; 0.81. Ratio of tibia to basitarsus for fore, mid and hind legs: 1.15; 1.57; 1.86.

Wing hyaline. Length of wing 4.00 – 4.23 mm (n=2). Ratio of length to width 2.83. Veins light brown, setose on both surfaces. Wing membrane with dense irregularly arranged microtrichia and with few macrotrichia in anal area and close to wing tip below of R1 and R5. C not produced beyond apex of R5, which is strongly sinuate. Sc ends in C at the level of beginning of medial fork. Sc2 situated at the level of middle of bM-Cu. M1basally obsolete. Cubital fork begins slightly before the base of r-m. Haltere pale with brownish knob. Both, stem and knob with short setae.

Abdomen brown with segments I–IV lighter. Terminalia (Figs 3a, 3b, 3c) light brown. Cercus distinctly two-segmented, segments with subequal length, proximal segment more than twice as wide as distal. Tergite VIII subquadrate, as large as tergite IX and tergite X together. Sternite VIII with deep ventral cleft. Gonapophysis IX well sclerotized and visible in lateral view. Hypoproct with apical incision, exposed in ventral view.

Male (Figs 2b, 3d).

Length of body 5.36 – 6.32, 5.65 mm (n=5). Length of wing 4.37 – 5.30, 4.76 mm (n=5), ratio of length to width 2.55 – 2.82, 2.64 (n=5). Coloration and other non-terminal characters similar to female except anepisternum, which has short setae on upper part. Terminalia brown. Gonostylus simple, without any additional lobes or spines, slender, tapering, curved medially and covered with short setae.

## Conclusive remarks

Vockeroth (1972) described *Baeopterogyna nudipes* in both sexes. In contrast to the normally developed wings in male specimens, the wings in females were greatly reduced: narrow and extending only to the apex of second abdominal tergite. Female halteres were also reduced, with a weak knob (cf. Vockeroth 1972: Figs 2, 5, 6). As the description of females based on two specimens from separate samples (although from the same locality: Herschel Island in Yukon Territory, Canada), this peculiarity cannot be argued as a possible aberration. Hackman (1964) summarized the knowledge of wing reduction in Diptera and discussed, among others, low temperature and wind-exposed habitats like oceanic islands as the causes. In addition to *Baeopterogyna nudipes* from an arctic habitat, only a few fungus gnats (Sciaroidea excl. Sciaridae) are described with reduced wings. *Macrocera crozetensis* Colless, 1970 with stenopterous females (cf. Matile 1975b: Figs 2, 3) has been described from the subantarctic Crozet Islands and *Mycetophila brachyptera* Duret, 1989 with two forms, one of them brachypterous (cf. Duret 1989: Figs 9 – 15), has been described from cold and wet forest of the Argentine-Chilean Patagonia. *Moriniola grilloti* Matile, 1976 with reduction of wing width in females (cf. Matile 1976: Fig. 1) has been described from the Afrotropics (Mayombe, Congo). In most cases, the reduction is obviously caused by the extreme habitat in which the species lives and is a characteristic only of females. This is an adaptive response to environmental pressure and can provide fitness advantages as shown in female flightlessness of some geometrid moths (e.g. Wahlberg et al. 2010). This type of reduction cannot be expected in congeners that inhabit more favourable habitats and finding *Baeopterogyna mihalyii* with normally developed wings of both sexes in central Europe is therefore not surprising.

## Supplementary Material

XML Treatment for Baeopterogyna mihalyii
